# Unveiling the psychosocial impact of Elexacaftor/Tezacaftor/Ivacaftor therapy in Cystic Fibrosis patients

**DOI:** 10.1186/s12890-024-03455-2

**Published:** 2025-02-17

**Authors:** Marta Solís García, Adrián Peláez, Rosa Mar Gómez Punter, María Criado López, Claudia Madrid Carbajal, Julio Ancochea, Jose María Eiros Bachiller, Ana Sofía Martín Hernández, María Rodrigo-García, Marta García Clemente, Rosa Mª Girón Moreno

**Affiliations:** 1 Servicio de Neumología, Instituto de Investigación La Princesa, Madrid, Spain; 2 Facultad de Ciencias de la Salud-HM Hospitales, Universidad Camilo José Cela, Madrid, Spain; 3 Centro de Investigación en Red de Enfermedades Respiratorias (CIBERES), Instituto de Salud Carlos III (ISCIII), Madrid, 28015 Spain; 4 Servicio de Neumología. Hospital Universitario Central de Asturias. ISPA (Instituto de Investigación del Principado de Asturias), Asturias, Spain; 5 Facultad de Medicina, Universidad Autónoma de Madrid, Madrid, Spain

**Keywords:** Cystic fibrosis, CFTR modulators, Lung function, Quality of life, Health mental, Anxiety and depression

## Abstract

**Background:**

This study aimed to assess how Elexacaftor/Tezacaftor/Ivacaftor (ETI) influences lung function, Body Mass Index (BMI), Sweat Test (ST) and mental health of Cystic Fibrosis (CF) patients, emphasizing on depression and anxiety.

**Methods:**

We conducted an observational, prospective, multicentre study including 108 patients over 18 years old who initiated ETI therapy between December 2019 and December 2023. Patients underwent regular evaluations, including clinical, functional, and microbiological assessments, alongside completion of quality of life, anxiety, and depression questionnaires. We evaluated whether there was a difference in anxiety and depression levels over time.

**Results:**

After 12 months of treatment, significant improvements were noted in BMI, lung function (FEV1%), ST and various aspects of quality of life (CFQ-R). However, anxiety and depression levels did not differ significantly during the follow-up. When we stratified our sample by key groups, we observed that younger patients (under 28 years) and those with homozygous Phe508del mutations experienced significant higher anxiety with no differences on depression. Furthermore, anxiety and depression demonstrated a moderate correlation, strengthening over time.

**Conclusions:**

Treatment with ETI establishes significant improvements in lung function, BMI, ST and quality of life in patients with CF. However, despite these positive outcomes, there were no significant changes observed in levels of anxiety and depression, except for individuals with homozygous mutation type and those younger than 28 years old, who exhibited significant higher levels of anxiety.

**Supplementary Information:**

The online version contains supplementary material available at 10.1186/s12890-024-03455-2.

## Introduction

Cystic fibrosis (CF) is a genetic disease with multiorgan involvement based on dysfunction or absence of the cystic fibrosis transmembrane conductance regulator (CFTR) protein, which constitutes a chloride channel in the secretory epithelial cells of the digestive and respiratory system, sweat glands, and genital system [1]. Patients with CF have traditionally been complex cases, with progressive decline in lung function, frequent exacerbations, microbiological isolates, usual pancreatic involvement, and repeated hospitalizations with a very limited life expectancy. Since the advent of highly effective modulator therapy (HEMT) with Elexacaftor/Tezacaftor/Ivacaftor (ETI), the paradigm of the disease has undergone an unprecedented shift, with clear improvements already observed in lung function, body mass index (BMI), exacerbations, microbiological isolates, and ultimately, quality of life [2].

However, the impact of CF doesn’t just affect individuals on a physical level; it also profoundly impacts mental health as patients grappling with CF often find themselves contending with high levels of depression and anxiety. This psychological toll underscores the complex nature of CF, highlighting the need for comprehensive support systems and interventions that address both the physical and mental well-being of those affected. Consequently, the Cystic Fibrosis Foundation and the International Committee on Mental Health in Cystic Fibrosis recommend annual depression and anxiety screenings using the Patient Health Questionnaire-9 (PHQ-9) and the Generalized Anxiety Disorder Scale-7 (GAD-7) [3].

The observational and experimental studies conducted to date with ETI have focused on evaluating objective data such as forced expiratory volume in first second (FEV1), forced vital capacity (FVC), BMI, exacerbations, and microbiological isolations, yielding very positive results [4–6]. However, they have not yet included outcomes pertaining to the psychosocial sphere of the patients, as the concepts of “anxiety” and “depression” are not encompassed in the pivotal clinical trials of the drug. Therefore, despite the previously mentioned benefits of ETI, the evolution of the mental health of patients with CF is still an enigma.

Since the approval of CFTR modulators in the United States in 2019, case series have emerged, indicating a concerning trend: some patients may experience deteriorating mood, sleep problems, anxiety, and an uptick in suicide attempts upon commencing treatment with ETI [7–11]. This revelation adds a layer of complexity to the management of CF, urging healthcare providers to remain vigilant and proactive in monitoring and addressing not only the physical symptoms but also the mental health aspects of CF treatment.

Considering these findings, a critical update has been incorporated into the technical information for ETI. The addition includes a crucial warning: “*Depression (including suicidal ideation and suicide attempts) has been reported in patients treated with ETI*,* which typically occurs within three months of starting treatment and in patients with a history of psychiatric disorders. In some cases*,* symptom improvement was reported after dose reduction or discontinuation of treatment. Patients (and their caregivers) should be advised to monitor for the onset of depressive moods*,* suicidal thoughts*,* or unusual changes in behaviour*,* and to seek immediate medical attention if these symptoms occur*”. This vital precaution highlights the importance of proactive patient monitoring and the urgent need for swift medical intervention in cases where mental health concerns arise during ETI treatment.

In contrast, there are other studies indicating psychological improvements after initiating ETI. For instance, a study by Martin et al.. demonstrated how patients with CF experienced enhancements in physical, psychological, and social aspects after starting the new treatment. This led to a higher quality of life and the development of new personal aspirations [12].

As we navigate this new chapter in CF care, it’s necessary to prioritize comprehensive patient support and foster open dialogue to mitigate potential risks and optimize outcomes. Given the complexities outlined above, it becomes evident that additional research is warranted to comprehensively understand the psychosocial impacts accompanying the initiation of ETI. Our study aimed to provide valuable insights into how ETI influences both mental health and associated physiological parameters, thus guiding future therapeutic strategies, and optimizing patient care.

## Materials and methods

**Fig. 1:**
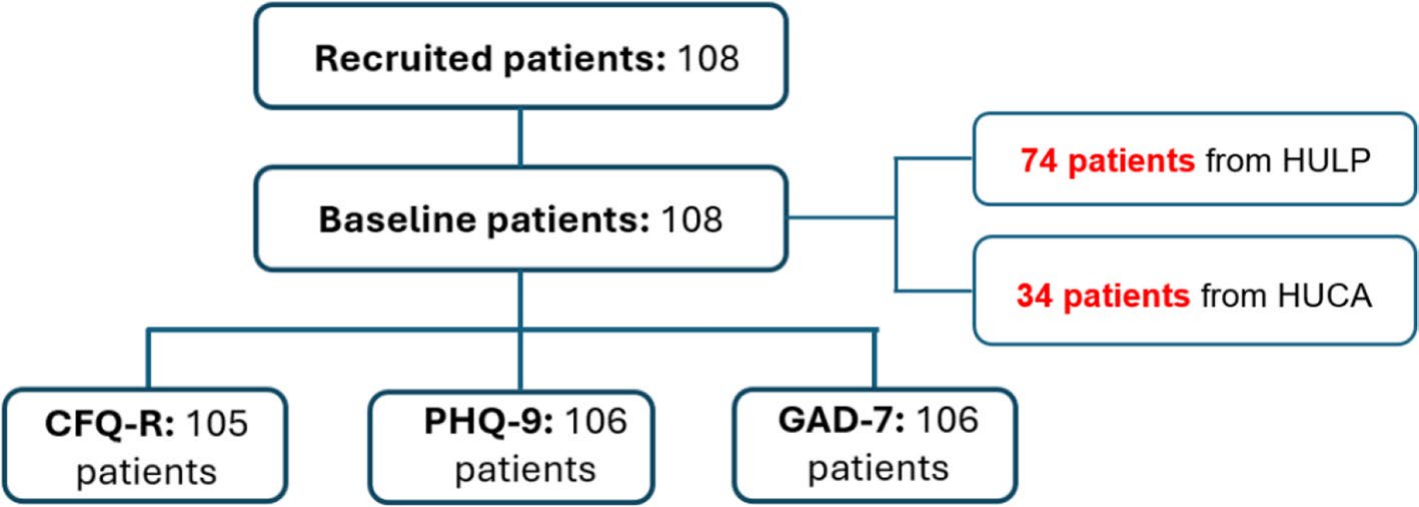
Flow chart of recruited patients with CF. CFQ-R: Cystic Fibrosis Questionnaire-Revised, GAD-7: Generalized Anxiety Disorder Scale-7 Items, PHQ-9: Patient Health Questionnaire-9. HULP: La Princesa University Hospital, HUCA: Central University Hospital of Asturias

We conducted an observational, prospective, multicentre study. We included as a selection criterion, patients over 18 years of age who were regularly followed up in the clinic and had started ETI between December 2019 and December 2023. In total, our study comprised 108 patients (Fig. 1) from two CF units in the country: La Princesa University Hospital (74 patients) and Central University Hospital of Asturias (34 patients).

The study was approved by the Clinical Research Ethics Committee (CEIm) of both hospitals (CEIm Ref No *957/2020* and *067/2020*), and informed consent was obtained from all included patients to participate in this study.

The patients were seen in the specific CF clinic every 3 months, being evaluated from a clinical, functional, and microbiological perspective at each visit.

For each patient, demographic (date of birth, sex, age at the beginning of the treatment and race) and anthropometric (height, weight and BMI) data was collected. We also obtained the sweat test results of patients from one of the two units included in the study, both before and after the start of ETI (the brand name of the equipment was Macroduct Advanced, Bigen Diagnostica SL). Genetic analysis was performed upon diagnosis of the disease, initially by analysis of frequent pathogenic variants (specific kits, including poli-T and poli-TG). In cases where none of these variants were found, gene sequencing of CFTR was done.

To monitor pulmonary function, we used basic spirometry in all visits, obtaining FVC, FEV1 and FEV1/FVC ratio, comparing the results with the reference values for the same weight, height, age, sex and race (Spirometer brands: ELITE SERIES TM, Medgraphics and CPFS/D USB™ Spirometer - MGC Diagnostics). From a microbiological point of view, sputum cultures for bacterial, mycobacterial and fungi were collected and analysed prior to each visit, counting as an exacerbation any episode of worsening of the basal state that from medical criteria, required antibiotherapy, whether oral or intravenous, and distinguishing between those that required hospitalization for more than 24 h and those that did not.

Additionally, the specific Cystic Fibrosis Quality of Life Questionnaire (CFQR) scores for respiratory, digestive, physical activity, role, eating disturbances, treatment, vitality, and emotional aspects were collected both before and after starting treatment, also every 3 months. Each section can reach a maximum score of 100 points, indicating better quality of life [13].

The assessment of patients’ psychomorbidity was conducted by reviewing scores from the PHQ-9 (a 9-item depression questionnaire with scores ranging from 0 to 27) and GAD-7 (a 7-item anxiety questionnaire with scores ranging from 0 to 21) [14, 15].

Depression severity was classified as normal/minimal, moderate, moderately severe, or severe based on PHQ-9 scores (0–4, 10–14, 15–19, ≥ 20, respectively), while anxiety severity was categorized as normal/minimal, mild, moderate, moderately severe, or severe, corresponding to GAD-7 scores (0–4, 5–9, 10–14, and ≥ 15, respectively).

**Fig. 2:**
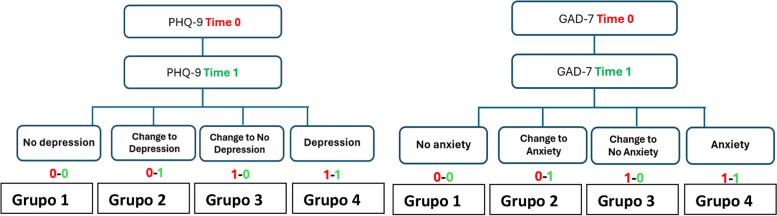
Distribution of patients into four distinct groups based on their PHQ-9/GAD-7 scores at baseline and final study time

As a general vision, for any of the two questionnaires, a score below 5 points was considered “No depression/anxiety,” while a score of 5 points or higher was considered “Depression/anxiety”. Taking this into account, for each condition (depression or anxiety), patients were grouped into four categories based on changes between pre- and post-ETI (0 and 12 months respectively): Group 1 (no depression/anxiety), Group 2 (newly developed depression/anxiety), Group 3 (improved depression/anxiety), and Group 4 (persistent depression/anxiety), as shown in Fig. 2.

Regarding the statistics, a preliminary descriptive analysis of patient characteristics was conducted by computing measures of central tendency and dispersion for quantitative variables and counts and percentages for qualitative variables. The normality and homoscedasticity of continuous variables were assessed using the Shapiro-Wilk and Kolmogorov-Smirnov tests, and Levene’s test, respectively. Parametric tests were employed when data distributions were both normal and homoscedastic, while non-parametric tests were utilized when these assumptions were not met. For qualitative variables, comparisons of proportions were assessed using either the χ2 test or Fisher’s exact test, as appropriate. Median differences were conducted and their Confidence Intervals (CI) using a bootstrap of 9999 replicates. For the cut-off points for FEV1 and age, the median for both values in our sample was used. Inter-rater agreement to measure outcomes was assessed using intraclass correlation (ICC) analysis. In any comparison, a value of *p* < 0.05 was considered statistically significant. All data management, statistical computations, and graphical representations were carried out using the R statistical software.

## Results

### Demographic and clinical characteristics before starting HEMT

The sociodemographic and clinical characteristics of 108 CF patients are shown in Table 1. The median age was 30.0 years [17.0–60.0], with a slight predominance of males (54.6%). Regarding pancreatic involvement, 84.3% had exocrine pancreas issues, while 41.7% exhibited endocrine pancreas complications. Hepatopathy was observed in 24.1% of the total cohort. In terms of infections, the most presented microorganism was Methicillin-sensible *Staphylococcus aureus* (MSSA) (58.3%) followed by *Pseudomonas aeruginosa* (36.1%). Notably, non-invasive mechanical ventilation was not required by any participant, and only a small percentage (6.5%) received oxygen therapy. The utilization of treatments revealed that 42.6% of the total cohort had received a previous modulator, being *Tezacaftor and Ivacaftor* the most common, administered to 89.1% of them. Side effects were generally low (5.6%). Regarding transplant-related outcomes, only a small percentage of the population was on the transplant active list (1.9%) although a slightly bigger group (5.6%) had been evaluated by the transplant unit team. No participants were reported as deceased during the study period.

**Table 1:** Sociodemographic and clinical characteristics of CF patients at the beginning of the treatment

**Patient characteristics at baseline (*N*=108)**	
Age (years)	30.0 [17.0, 61.0]
Sex [Male]	59 (54.6%)
Type of mutation [ Heterozygous]	59 (54.6%)
Exocrine pancreas [pancreatic insufficiency]	91 (84.3%)
Endocrine pancreas [hydrocarbon intolerance]	45 (41.7%)
Hepatopathy	26 (24.1%)
FEV1 (%)	63.5 [29.0, 114.0]
FVC (%)	80.5 [43.0, 119.0]
BMI (kg/m2)	21.7 [16.2, 32.0]
**Infections at baseline**	
Methicillin-sensible *Staphylococcus aureus*	63 (58.3%)
*Pseudomonas aeruginosa*	39 (36.1%)
Methicillin-resistant *Staphylococcus aureus*	9 (8.3%)
*Haemophilus influenzae*	6 (5.6%)
Non-tuberculous Mycobacterium	5 (4.6%)
Other	40 (37.0%)
**Previous Treatments**	
Previous Modulator	46 (42.6%)
*Ivacaftor*	2 (4.3%)
*Tezacaftor and Ivacaftor*	42 (91.4%)
*Lumacaftor and Ivacaftor*	2 (4.3%)
Age at Previous Modulator (years)	27.0 [15.0, 45.0]
**Use of ETI**	
Age at ETI start (years)	28.0 [12.0, 59.0]
Side Effects after ETI	6 (5.6%)
Cholecystopancreatitis	1 (16.6%)
Hepatic	2 (33,4%)
Rash	3 (50.0%)
Suspension of ETI	5 (4.6%)
Out of Stock	1 (20.0%)
Side Effect	4 (80.0%)
Transplant Unit Evaluation pre-ETI	6 (5.6%)
Transplant Active List pre-ETI	2 (1.9%)
**Questionnaires**	
**GAD7**	
Normal [score: 0–4]	67 (62.0%)
Mild [score: 5–9]	27 (25.0%)
Moderate [score: 10–14]	7 (6.5%)
Severe [score: 15–21]	5 (4.6%)
**PHQ9**	
Normal [score: 0–4]	67 (62.0%)
Mild [score: 5–9]	25 (23.1%)
Moderate [score: 10–14]	12 (11.1%)
Moderately severe [score: 15–19]	1 (0.9%)
Severe [score: >20]	1 (0.9%)

Data are shown as n (%), median [minimum, maximum]

### Depression, anxiety and quality of life

The difference in medians for the variables were conducted comparing baseline with the results at 3, 6, 9, and 12 months from the start of ETI (Table 2). Improvement for BMI, FEV1%, and the respiratory, role, and emotional domains of CFQ-R began to be visible and statistically significant as early as the first 3 months after the initiation of treatment, an improvement that also remained statistically significant after completing one year of treatment. For the digestive, vitality, and eating domains of CFQ-R, significant improvement was observed at 12 months, with this difference not being statistically significant earlier. In the case of the PHQ-9 questionnaire for depression, a punctually significant improvement was observed at 9 months. For the treatment domain of the CFQ-R and the GAD-7 questionnaire for anxiety, no significant difference in medians was observed during the whole year of follow-up.

**Table 2:** Effects of ETI on quality of life determined by the CFQ-R

ITEM	Change between baseline and ETI 3 Months (CI_95%_)^1^		Change between baseline and ETI 6 Months (CI_95%_)		Change between baseline and ETI 12 Months (CI_95%_)		
**CFQ-R Respiratory**	27.77 [ 16.67–27.77]	***p*< 0.001**	27.77 [ 16.51–27.77]	***p*< 0.001**	27.77 [ 11.11–27.77]	***p*< 0.001**	
**CFQ-R Digestive**	0.00 [−11.00– 0.001]	*p* = 0.299	11.10 [−11.11–11.11]	*p* = 0.247	11.10 [−1.33–11.11]	***P*= 0.003**	
**CFQ-R Vitality**	−8.33 [−16.66–8.33]	*p* = 0.830	0.00 [−8.33 − 8.33]	*p* = 0.691	11.10 [−4.35 − 11.11]	***p*= 0.003**	
**CFQ-R Physical activity**	16.66 [8.33–20.83]	***p*< 0.001**	16.66 [7.61–20.83]	***p*< 0.001**	25.00 [16.66 − 29.17]	***p*< 0.001**	
**CFQ-R Food**	11.11 [0.00–11.12]	*p* = 0.025	11.11 [−11.12–11.12]	*p* = 0.117	11.11 [ 0.00–11.12]	***p*= 0.007**	
**CFQ-R Diary activity**	8.34 [−8.34–8.34]	***p*< 0.001**	8.34 [−8.33–8.34]	***p*= 0.004**	8.34 [0.00–8.34]	***p*< 0.001**	
**CFQ-R Treatment**	0.00 [−11.11–0.00]	*p* = 0.594	0.00 [−11.11–0.00]	*p* = 0.841	0.00 [0.00–0.00]	*p* = 0.926	
**CFQ-R Emotional**	0.00 [−13.33–6.67]	***p*= 0.016**	3.33 [−6.67–6.66]	***p*= 0.002**	13.33 [6.66–20.00]	***p*< 0.001**	
**GAD-7**	−2.00 [−4.00 – −2.00]	*p* = 0.0876	−1.00 [−3.00–0.00]	*p* = 0.381	−1.00 [−3.00–0.00]	*p* = 0.434	
**PHQ-9**	−0.50 [−2.00–1.00]	*p* = 0.160	−1.50 [ −4.00 – −1.00]	*p* = 0.141	−0.50 [−2.00–1.00]	*p* = 0.193	

^1^Confidence Interval 95% using a boostraping method (*n* = 9999)

Significant comparisons (*p* < 0.05) are marked in bold

**Fig. 3:**
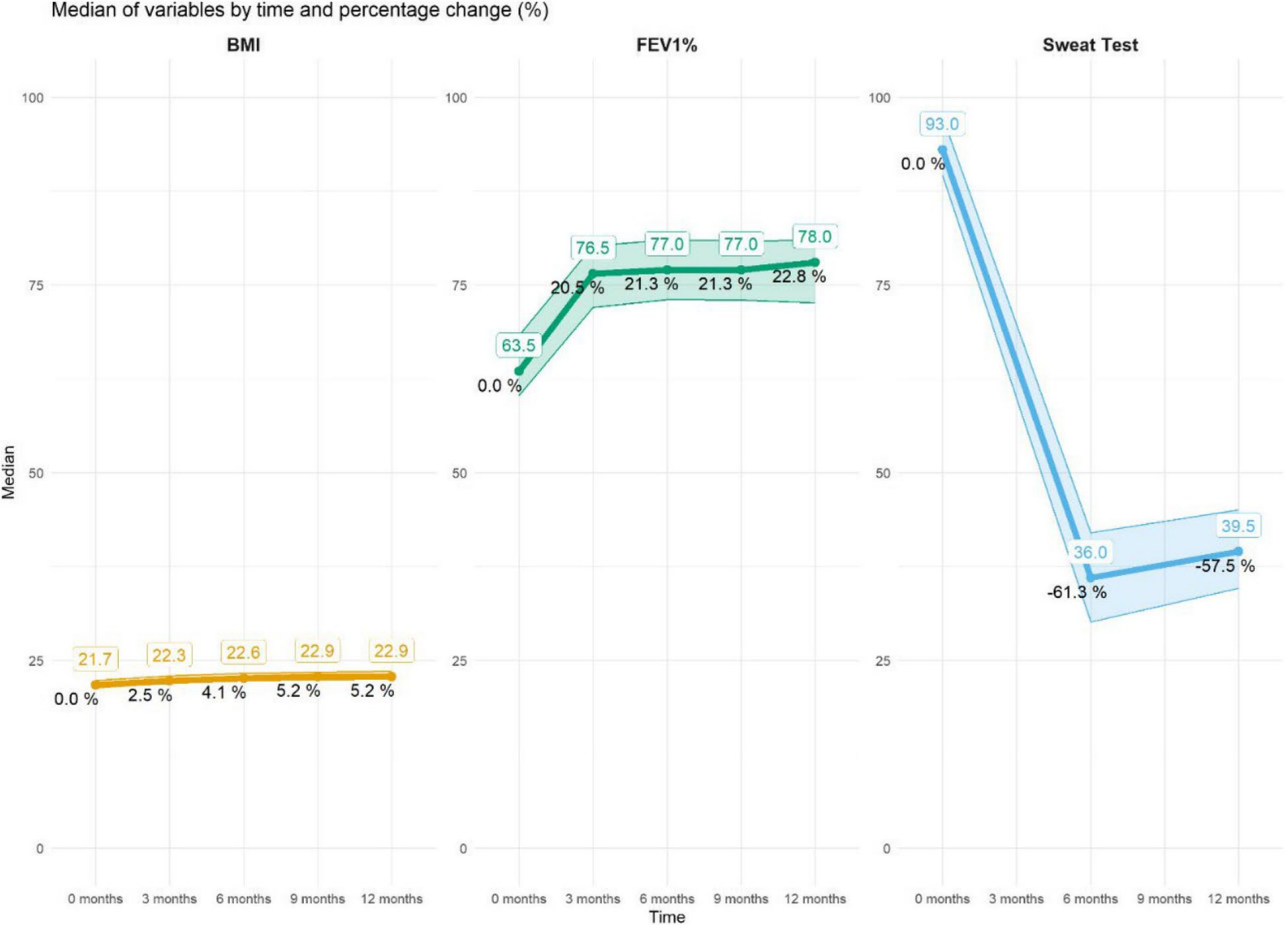
Evolution of the variables FEV1%, BMI, and sweat test at 3, 6, 9, and 12 months after starting HEMT. * The sweat test was only performed on the 34 patients from HUCA

We conducted median differences between the 12-month treatment period and baseline scores for the respiratory domain of the CFQR, as well as the PHQ-9 and GAD-7 questionnaires (Table 3; Fig. 3). This analysis was stratified by key factors such as sex, mutations, age (greater or less than the median age value), FEV1 status (greater or less than the median FEV1 value), and the presence of *Pseudomonas aeruginosa*. Significant differences in median scores were found across all subgroups when comparing the baseline CFQ-R respiratory domain scores with those after 1 year of treatment. Conversely, no significant differences were detected within any subgroup for the GAD-7 scores although PHQ-9 questionnaire showed a significant difference in median differences among Phe508del homozygotes and individuals younger than 28 years old.

**Table 3:** Assessment of ETI effects on CFQ-R respiratory domain, PHQ-9 and GAD-7, stratifying by key factors

Questionaire	Change between baseline and ETI median (CI_95%_)^1^	*p*-value	Change between baseline and ETI median (CI_95%_)	*p*-value	
**Groups**	**Female**		**Male**		
CFQ-R respiratory domain	27.77 [5.56–27.77]	**< 0.001**	22.22 [5.56–22.22]	**< 0.001**	
PHQ-9	0.0 [−2.80–0.00]	0.330	−0.5 [−2.00–0.00]	0.067	
GAD-7	0.0 [−2.00 – −1.00]	0.420	−1.0 [−3.00–0.00]	0.191	
**Groups**	**Heterozygous F508del**		**Homozygous F508del**		
CFQ-R respiratory domain	27.77 [11.11–27.77]	**< 0.001**	22.2 [5.56–27.77]	**< 0.001**	
PHQ-9	−1.0 [−3.00 – −1.00]	0.536	−1.0 [−3.00–0.00]	**0.020**	
GAD-7	−1.0 [−3.00 – −1.00]	0.538	−2.0 [−4.00– −2.00]	0.098	
**Groups**	**Median age < 28 years**		**Median age ≥ 28 years**		
CFQ-R respiratory domain	27.77 [ 11.11–27.77]	**< 0.001**	27.77 [16.66–27.77]	**< 0.001**	
PHQ-9	−1.5 [−3.00–0.00]	**0.020**	−0.5 [−2.00–1.50]	0.569	
GAD-7	−1.0 [−3.00 – −0.50]	0.098	−1 [−3.50–0.00]	0.605	
**Groups**	**Median FEV1% <63.5**		**Median FEV1% ≥63.5**		
CFQ-R respiratory domain	27.77 [16.66–33.33]	**< 0.001**	27.77 [11.11–27.77]	**< 0.001**	
PHQ-9	−0.5 [−2.00–1.00]	0.211	−1.5 [−3.50 – −0.50]	0.120	
GAD-7	−1.0 [−3.00– −1.00]	0.183	−0.5 [−2.00–1.50]	0.362	
**Groups**	***Pseudomonas aeruginosa*** **absence**		***Pseudomonas aeruginosa*** **presence**		
CFQ-R respiratory domain	27.77 [11.11–27.77]	**< 0.001**	27.77 [ 11.11–27.77]	**< 0.001**	
PHQ-9	−1.0 [−3.00–0.00]	0.053	−1.00 [−3.00–0.00]	0.403	
GAD-7	−1.0 [−2.50 – −0.50]	0.079	0.0 [−2.00–1.00]	0.800	

^1^Confidence Interval 95% using a boostraping method (*n* = 9999)

Significant comparisons (*p* < 0.05) are marked in bold

### Changes in depression based on PHQ-9 questionnaire

Prior to the initiation of ETI, our analysis revealed that out of 106 patients, the majority (63.2%), reported either no or minimal depression (scored 0–4 points). Mild depression (scored 5–9 points) was reported by 23.6% (25 patients) of patients, while 11.3% (12 patients) reported moderate depression (scored 10–14 points). The incidence of moderate-severe depression (scored 15–19 points) was minimal, with only 0.9% (1 patients) of patients affected, along with 0.9% reporting severe depression characterised by a questionnaire score exceeding 20 (Table 1).

**Fig. 4:**
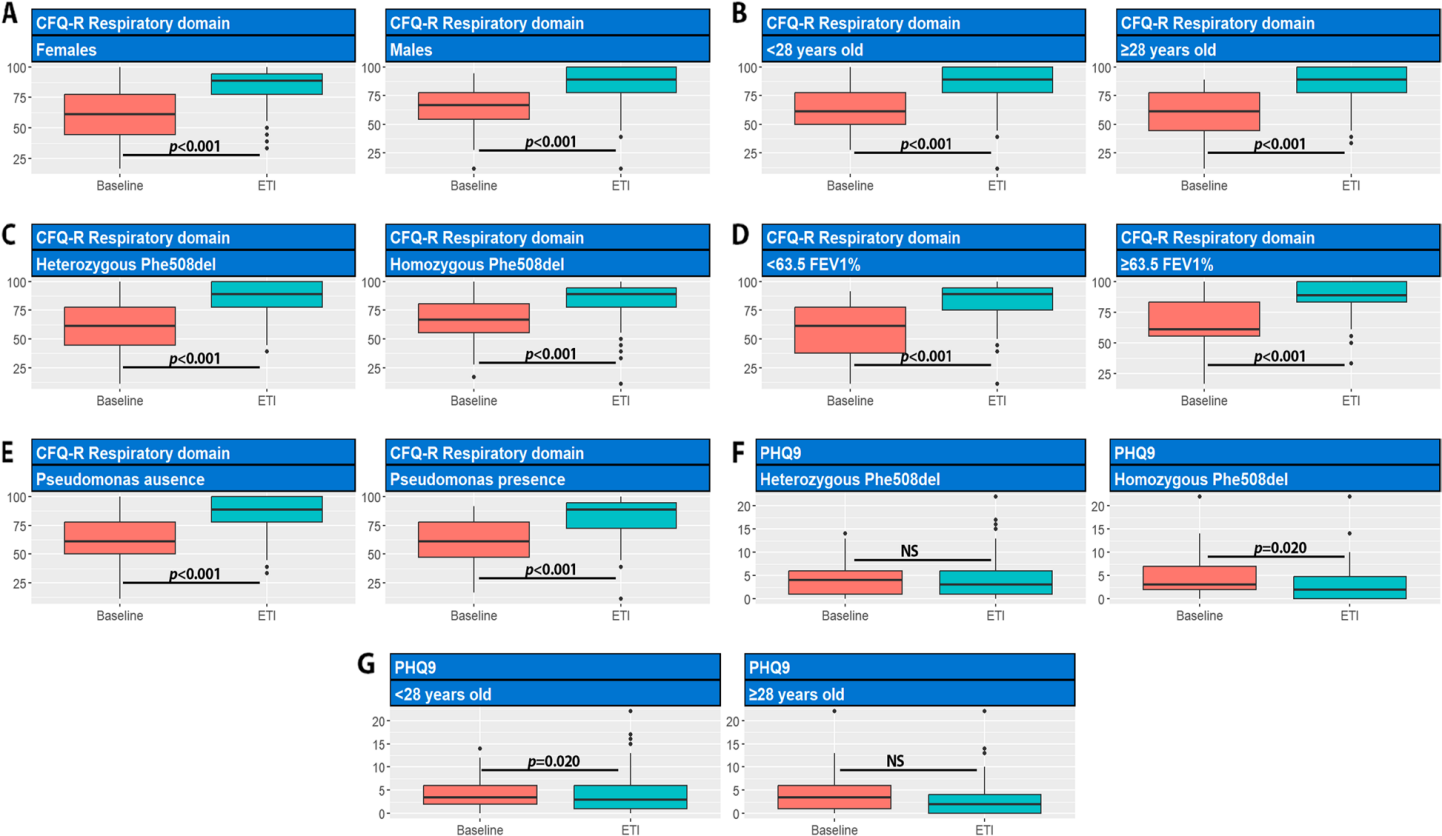
Paired assessment for effects of ETI on CFQ-R domains and PHQ-9 in total and depending on sex, age, mutation, lung function, presence of *Pseudomonas aeruginosa* (including only groups that showed unless one significant difference between groups)

After 12 months of therapy, 13 patients were lost to follow-up. Upon analysing the questionnaires collected from the remaining 93 patients, a slight increase in the percentage of patients without depression was observed, rising to 66.7% (62 patients). Additionally, the proportion of patients reporting mild depression slightly increased to 24.7% (23 patients). Notably, the percentage of patients reporting moderate depression rose to 7.5% (7 patients), while severe depression remained limited to 1.1%, affecting a single patient (Fig. 4).

Baseline characteristics of the groups were analysed in relation to the development of depression assessed by the PHQ-9 questionnaire over the course of the study as shown in Fig. 2. A notable observation was the predominance of females in Group 2, the cohort that developed depression. Additionally, the median age at the start of ETI for Group 3, the cohort that transitioned to no depression, was 24.5 years, slightly lower than the other groups. In terms of pulmonary function, groups 3 and 4 exhibited slightly depressed baseline FEV1% with medians of 58% and 54% respectively, indicating potential respiratory impairment in these groups (Table 5 in Appendix). Regarding microbiological isolations, 58.3% of patients in Group 2 had chronic *Pseudomonas aeruginosa* infection. However, none of these observed differences reached statistical significance.

**Fig. 5:**
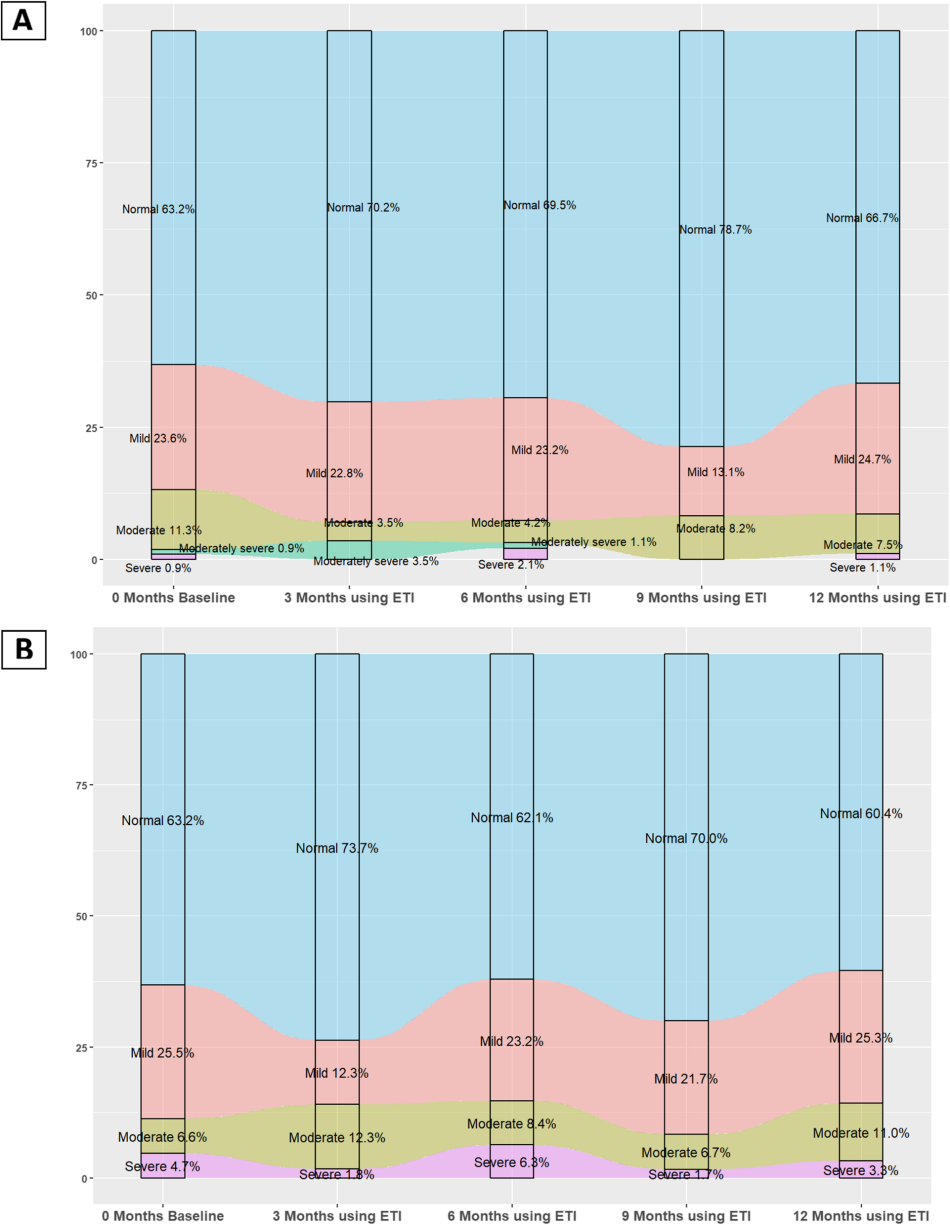
Alluvial plot depicting the distribution of patients during the follow-up of ETI therapy of the categories for Patient Health Questionnaire-9 (PHQ-9) (**A**) and Generalised Anxiety Disorder-7 (GAD7) (**B**)

### Changes in anxiety, based on GAD-7 questionnaire

When we examined the anxiety levels of our patients prior to initiating ETI therapy (106 individuals), we found that the majority experienced either no anxiety or minimal anxiety (scored 0–4 points), with 63.2% of our sample (67 patients) falling into this category. Mild anxiety (scored 5–9 points) was reported by 25.5% (27 patients), while 6.6% (7 patients) reported moderate anxiety (scored 10–14 points), and only 5 patients (4.7%) reported severe anxiety, scoring above 15 on the questionnaire (Table 1).

Following 12 months of initiating modulatory therapy, 15 patients were lost to follow-up, leaving 91 participants who completed the GAD-7 questionnaire. Updated assessments indicated a decrease in individuals reporting no or minimal anxiety to 55 patients (60.4%) and a reduction in those experiencing severe anxiety to 3 patients (3.3%). The proportion of patients with mild anxiety remained consistent, with 23 individuals (25.3%) falling within this category. Notably, the number of patients exhibiting moderate anxiety increased to 10 (11%) (Fig. 5).

Baseline characteristics of the groups were analysed in relation to the development of anxiety assessed by the GAD-7 questionnaire over the course of the study as shown in Fig. 2. We observe a predominance of males (67.4%) in Group 1 (those who never had anxiety) with a predominance of females in the remaining groups. Regarding BMI, a higher index (23.2) is observed for Group 2 (those who did not have anxiety and developed it). In terms of respiratory function, no significant differences are observed, with similar mean FEV1% across all 4 groups (Table 6 in Appendix). None of the observed differences reached statistical significance.

### Evolution of pulmonary function between depression and anxiety along the study

**Fig. 6:**
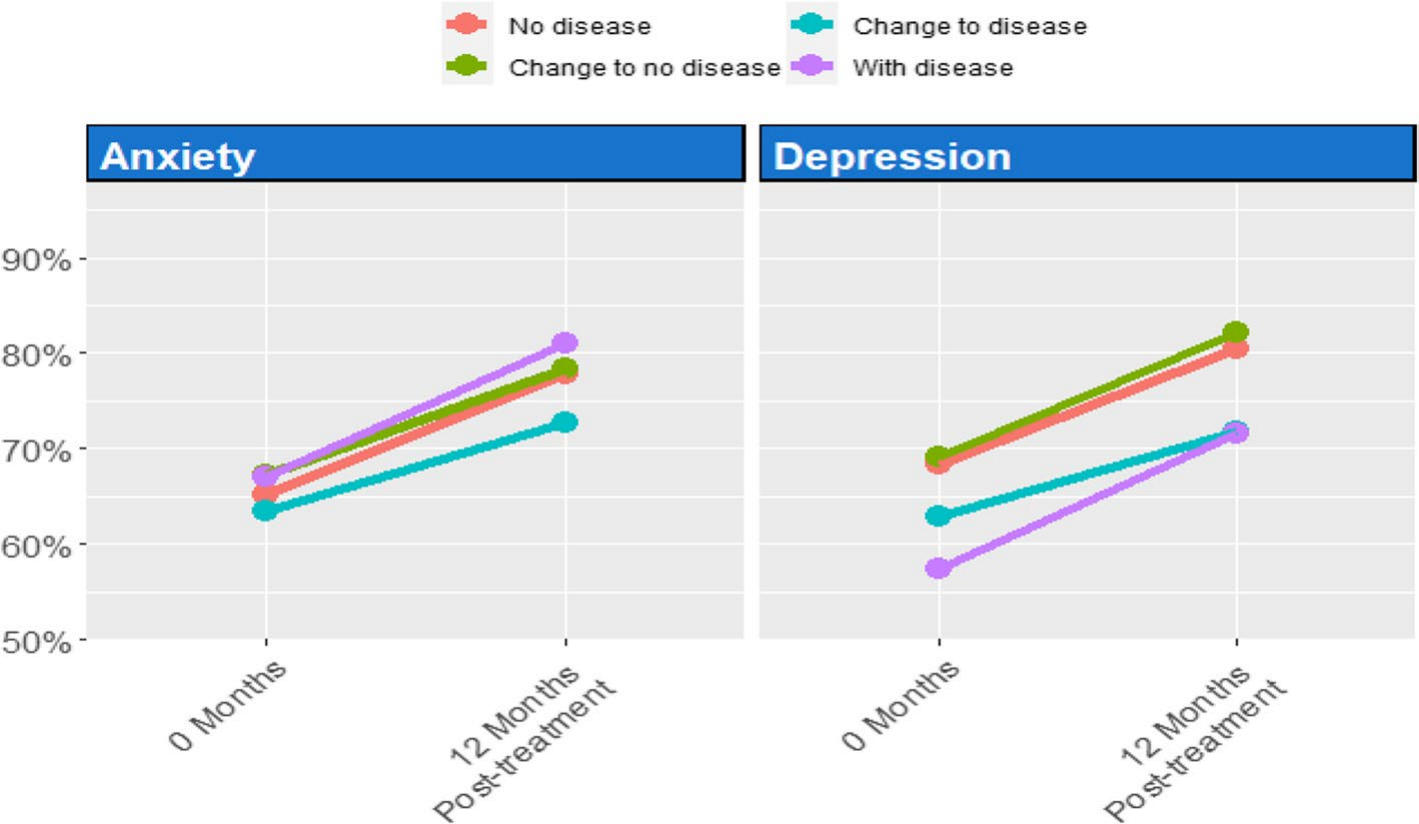
Evolution of FEV1 at baseline and 12 months post-treatment for the different groups according to the GAD-7 and PHQ-9 groups

Taking into account the established groups for PHQ-9 and GAD-7 (Fig. 2), the evolution of lung function measured in FEV1% was studied and depicted for each cohort in Fig. 6.

Regarding the GAD-7 anxiety questionnaire, Group 2 displayed diminished FEV1% in comparison to the other cohorts, corresponding to individuals who developed anxiety after treatment initiation. Nonetheless, no statistically significant differences were discerned.

As for the PHQ-9 questionnaire, diminished FEV1% was noted in Groups 2 and 4, denoting impaired pulmonary function in both those who experienced depression throughout the study period and those who developed it following the commencement of ETI. These results, while clinically meaningful, did not reach statistical significance.

### Correlation between anxiety and depression

The inter-agreement between anxiety and depression, in total and for each time point of the study, was measured through the analysis of Intraclass Correlation Coefficients (ICCs) (Table 4). The findings reveal a significant correlation between anxiety and depression throughout the study, with an overall ICC of 0.560 (*p* < 0.001), classified as moderate. Additionally, a trend of increasing correlation over time is observed, from a discrete correlation at 0 months (ICC = 0.370) to a substantial correlation at 6 months (ICC = 0.761). However, from 9 months onwards, this correlation remains moderate.

**Table 4:** Correlation between anxiety and depression throughout study

Groups	ICC^a^	LCI^b^	UCI^c^	Interval length	*p*-value	Classification
**Classification of Anxiety and Depression**						
Total	0.560	0.499	0.615	0.116	**< 0.001**	Moderate
0 Months	0.370	0.196	0.522	0.326	**< 0.001**	Discrete
3 Months	0.644	0.518	0.742	0.224	**< 0.001**	Substantial
6 Months	0.761	0.668	0.830	0.162	**< 0.001**	Substantial
9 Months	0.549	0.403	0.669	0.266	**< 0.001**	Moderate
12 Months	0.517	0.368	0.643	0.275	**< 0.001**	Moderate

^a^ Intraclass correlation coefficients

^b^ Lower confidence Interval

^c^ Upper confidence Interval

## Discussion

CF remains a complex and challenging disease to manage, requiring a multidimensional approach to treatment. Emerging therapies targeting the CFTR protein have raised hopes for improved outcomes in CF patients, but the evidence on mental health is still inconclusive. Our study, involving a relevant sample of CF patients over a one-year period, did not show statistically significant differences in the evolution of anxiety and depression levels following the start of therapy, although we did observe a significant correlation between both mental health conditions. This finding aligns with prior research [16–19], showing statistically significant improvements in lung function and quality of life as measured by the CFQ-R questionnaire, but inconclusive results in mental health outcomes.

Our results are consistent with the current literature, including the studies by Zhang et al. and Pudukodu et al., which also did not observe significant changes in anxiety and depression scores after initiating ETI therapy [20, 21]. Additionally, a comprehensive review from the University Of Washington School Of Medicine concluded that changes in depression may be more attributable to patients’ baseline characteristics rather than a direct effect of ETI [22].

However, our findings contrasts with other results, such as the ones revealed by Evelyne et al., who documented new neuropsychiatric symptoms in a subset of patients who started ETI, including cognitive disorders, insomnia, depression, anxiety, lack of energy, mania and hypomania [23]. On the other side, an Italian study, that analysed the psychiatric behaviour of 92 patients during the first 6 months after starting ETI, found improvements in depression during the first month post-therapy, but no change in anxiety levels [24]. These discrepancies highlight the complexity of evaluating mental health outcomes in CF patients and suggest that a range of external factors may influence results.

Although no statistically significant results were found concerning mental health in our study, we revealed a slight decrease on the prevalence of depression and a mild uptick in the percentage of patients who exhibited anxiety during the 12 months of follow-up. This findings highlight the dynamic nature of mental health in CF patients, probably related to new treatment routines, new expectations, or weight gain, as eating disorders are another major obstacle in CF patients [25, 26].

In a subgroup analysis, we found that younger patients (< 28 years) experienced higher levels of anxiety, possibly due to developmental factors or less effective coping mechanisms. Additionally, patients with a homozygous Phe508del mutation also exhibited greater anxiety, likely related to concerns about disease severity, prognosis, and the impact of treatment on daily life [27]. In contrast, depression scores did not show significant variation across demographic groups. These findings emphasize the importance of personalized psychological support for CF patients, tailored to their age and genetic background.

It is pertinent to highlight the potential relation between the occurrence of anxiety and a rise in patients’ BMI after the commencement of ETI (Anex 2) [28]. This association may be related to changes in body perception, as CF patients are often accustomed to maintaining lower weights despite high caloric intake. The notable rise in BMI following treatment initiation could potentially trigger higher anxiety levels, influenced by concerns about body image or the psychological impact of weight gain [29–31]. Further research is imperative to determine whether this relationship is causal or consequential. Specifically, future studies should explore how body image perception and associated psychological factors evolve over time in CF patients receiving ETI, and whether tailored support can mitigate these anxiety responses.

Despite these insights, our study is limited by the lack of formal psychological assessments and the absence of baseline psychiatric medication data. Additionally, the mental health outcomes did not correlate with clinical factors like exacerbations, hospitalizations, or treatment complexity. Nevertheless, our study stands out for its large sample size, long follow-up, and subgroup analyses, contributing valuable information to the limited literature on the mental health impact of ETI therapy.

CFTR modulator therapy holds promise for physiological improvements in CF patients but its impact on mental health remains a subject of ongoing investigation. Our study contributes to this discourse by highlighting the complexities and challenges of assessing mental health outcomes in the context of CFTR modulator therapy. Further research is needed to unravel the complex link between CFTR modulators and mental well-being.

## Conclusion

Our study demonstrated physiological benefits of triple therapy, including improved pulmonary function, BMI, and quality of life. However, the impact on mental health remains complex, and future research should focus on longitudinal, multidimensional analyses to better understand and address the psychosocial needs of CF patients. Holistic care models that integrate mental health assessments and support may optimize overall patient well-being.

## Supplementary Information

Supplementary Material 1.

## Data Availability

The datasets used and/or analysed during the current study are available from the corresponding author on reasonable request.

## Appendix

**Table 5:** Comparison of groups according to depression score between baseline and 12 months post-treatment

	No depression (*n*=47)	Change to non-depression (*n*=15)	Change to depression (*n*=12)	With depression (*n*=19)	*p*-value
Age	31.0 [17.0, 61.0]	30.0 [18.0, 49.0]	26.0 [18.0, 55.0]	32.0 [19.0, 48.0]	0.454
Sex [Male]	29 (61.7%)	7 (46.7%)	3 (25.0%)	10 (52.6%)	0.142
Heterozygous	26 (55.3%)	8 (53.3%)	8 (66.7%)	9 (47.4%)	0.797
BMI (kg/m^2^)	21.9 [16.2, 32.0]	21.8 [17.4, 24.5]	21.5 [18.5, 29.4]	21.2 [18.1, 25.3]	0.450
FEV1%	67.0 [29.0, 108]	71.0 [38.0, 114]	58.0 [30.0, 93.0]	54.0 [31.0, 102]	0.157
Comorbility					
Pancreatic insufficiency	39 (83.0%)	14 (93.3%)	10 (83.3%)	15 (78.9%)	0.771
Hydrocarbon intolerance	19 (40.4%)	8 (53.3%)	5 (41.7%)	5 (26.3%)	0.455
Hepatopathy	11 (23.4%)	5 (33.3%)	3 (25.0%)	3 (15.8%)	0.696
Isolations					
* Pseudomonas aeruginosa*	18 (38.3%)	4 (26.7%)	7 (58.3%)	6 (31.6%)	0.353
Treatments					
Previous Modulator	21 (44.7%)	6 (40.0%)	2 (16.7%)	11 (57.9%)	0.156

**Table 6:** Group comparison according to anxiety score between baseline and 12 months post-treatment

	No anxiety (*n*=43)	Change to no anxiety (*n*=12)	Change to anxiety (*n*=14)	With anxiety (*n*=22)	*p*-value
Age	30.0 [17.0, 58.0]	34.0 [23.0, 61.0]	29.5 [18.0, 55.0]	27.0 [18.0, 48.0]	0.335
Sex [Male]	29 (67.4%)	5 (41.7%)	6 (42.9%)	9 (40.9%)	0.108
Heterozygous	24 (55.8%)	3 (25.0%)	9 (64.3%)	15 (68.2%)	0.094
BMI (kg/m^2^)	21.5 [16.2, 32.0]	22.8 [19.2, 26.7]	23.2 [18.3, 29.4]	21.3 [18.1, 25.3]	0.064
FEV1%	63.0 [29.0, 108]	66.5 [34.0, 105]	62.5 [42.0, 93.0]	66.5 [31.0, 114]	0.950
Comorbility					
Pancreatic insufficiency	37 (86.0%)	11 (91.7%)	10 (71.4%)	18 (81.8%)	0.495
Hydrocarbon intolerance	20 (46.5%)	7 (58.3%)	5 (35.7%)	5 (22.7%)	0.155
Hepatopathy	11 (25.6%)	4 (33.3%)	4 (28.6%)	3 (13.6%)	0.557
Isolations					
* Pseudomonas aeruginosa*	19 (44.2%)	3 (25.0%)	5 (35.7%)	8 (36.4%)	0.659
Treatments					
Previous Modulator	20 (46.5%)	7 (58.3%)	4 (28.6%)	7 (31.8%)	0.302

## Abbreviations

Marta Solís García: M. Solís García
Adrián Peláez: A. Peláez
Rosa Mar Gómez Punter: R.M. Gómez Punter
María Criado López: M. Criado López
Claudia Madrid Carbajal: C. Madrid-Carbajal
Julio Ancochea: J. Ancochea
Jose María Eiros Bachiller: J.M. Eiros Bachiller
Ana Sofía Martín Hernández: A.S. Martín-Hernández
María Rodrigo-García: M. Rodrigo-García
Marta García Clemente: M. García-Clemente
Rosa Mª Girón Moreno: R.M. Girón Moreno
