# Supplementary material for: Unveiling the psychosocial impact of Elexacaftor/Tezacaftor/Ivacaftor therapy in Cystic Fibrosis patients
Source: BMC Pulm Med. 2025 Feb 17;25:81. doi: 10.1186/s12890-024-03455-2 (PMC11831831; doi:10.1186/s12890-024-03455-2)
Supplement: Supplementary file 1 — Supplementary Material 1. [file 12890_2024_3455_MOESM1_ESM.pdf]

PATIENT INFORMATION SHEET

Study Title: *"Impact of triple therapy with tezacaftor/elexacaftor/ivacaftor on systemic inflammation, microbiology, exacerbations, and quality of life in patients with cystic fibrosis."*

The purpose of this letter is to invite you to participate in a research project being conducted at the Central University Hospital of Asturias. The primary goal of this study is to evaluate the real-life impact of triple therapy with tezacaftor/elexacaftor/ivacaftor, which you are about to start, in terms of clinical improvement, reduction in exacerbations, changes in microbiology, improvements in your quality of life, and reductions in systemic inflammation.

Cystic fibrosis (CF) is the most common and severe autosomal recessive genetic disease in the Caucasian population, affecting 75,000 people worldwide. The disease is caused by mutations in the gene that encodes the cystic fibrosis transmembrane conductance regulator (CFTR) protein, a chloride and bicarbonate channel expressed in the epithelial cells of mucin-producing organs. The ion transport defect results in a multisystemic disease that affects the lungs, pancreas, intestines, liver, and reproductive organs.

The survival of patients with CF has significantly improved in recent years due to care provided by specialized multidisciplinary teams, appropriate use of antibiotics, improved nutritional status, and adherence to respiratory physiotherapy treatments. Recently, the introduction of CFTR modulators has also led to significant improvements in symptoms, quality of life, and survival for these patients.

CFTR modulator treatment began a few years ago, initially restricted to certain mutations. More recently, phase 3 studies demonstrated the significant efficacy of the triple combination of elexacaftor/tezacaftor/ivacaftor in patients with at least one copy of the DF508 mutation. These studies showed improvements in sweat test results, lung function, nutritional status, and quality of life in both homozygous DF508 patients and heterozygous DF508 patients with a minimal function mutation. This triple combination has recently been approved by the Ministry of Health for use in patients aged 12 years or older with at least one copy of the DF508 mutation. Consequently, nearly 80% of adolescents and adults with CF are eligible for this treatment, and overall, 90% of CF patients could benefit from CFTR modulators.

Since you have been diagnosed with cystic fibrosis and carry at least one copy of the DF508 mutation, you are eligible to begin the treatment described above. Our aim is to collect real-world data on the benefits of this therapy and also to analyze potential side effects.

During your visits to the CF Unit, no additional procedures will be performed beyond those you usually undergo during routine visits. Clinical variables will be collected, you will be asked about any exacerbations and antibiotic cycles you have received, and you will undergo respiratory function tests as usual. A sputum sample will be collected for microbiological analysis, and a blood sample will be taken for routine blood work along with a study of systemic inflammation markers. Additionally, you will complete self-administered quality-of-life questionnaires to help us assess the impact of this treatment on your daily life activities.

What data will be collected in this study?

The data collected will include demographic information such as sex, age, and BMI. Prior to the start of triple therapy, clinical data, exacerbation history, and microbiological data will be recorded. You will also be invited to complete self-administered quality-of-life questionnaires during your visit. Additionally, sputum samples will be collected for microbiological analysis, and a blood sample will be drawn for routine blood tests and systemic inflammation marker analysis. Any leftover samples will be frozen for potential future studies.

We will follow your routine appointments at the CF Unit, and at 6 and 12 months after starting the treatment, we will collect demographic, exacerbation, and microbiological data again. You will also complete the quality-of-life questionnaires as you did at the baseline visit. We will conduct routine blood work and systemic inflammation marker analysis again, freezing any leftover samples for future research.

What are the risks of participating in this study?

No additional risks are anticipated due to the nature of this study, as the procedures you will undergo are the same as those routinely performed during your follow-up visits at the CF Unit of HUCA.

What if I decide not to participate?

Participation in this study is entirely voluntary. You may choose not to participate or withdraw your consent at any time without affecting your relationship with the healthcare staff or your treatment. There is no financial compensation for patients or researchers in this study.

Confidentiality

All your data and medical information related to your condition necessary for the study will be treated with absolute confidentiality by the research team, who will be the only ones with access to this information. Data collected for the study will be identified using a code and completely dissociated from any information that could identify you. At no time will personal or identifiable patient data be disclosed, even if the study results are published in scientific journals.

The research team sincerely appreciates your participation in this study.

INFORMED CONSENT

Study: "Impact of triple therapy with tezacaftor/elexacaftor/ivacaftor on systemic inflammation, microbiology, exacerbations, and quality of life in patients with cystic fibrosis."

I, ____________________________________, have been informed by Dr. ____________________________________, a collaborator in the aforementioned research project, and I declare that:

- I have read the information sheet provided to me.
- I have asked any questions I had about the study.
- I have received adequate answers to my questions.
- I have received sufficient information about the study. I understand that my participation in this research project is completely voluntary and that I can withdraw at any time without providing explanations and without it affecting my medical care. I understand that all data will be kept confidential.

The confidentiality of the data obtained for this research will be maintained at all times, and under no circumstances will the identity of the participants be disclosed, even if the study results are published. The project data will be handled in strict compliance with the Personal Data Protection Law (Organic Law 3/2018, of December 5, on the Protection of Personal Data and the guarantee of digital rights (BOE-A-2018-16673)).

Consequently, I give my consent for the collection and preservation of biological fluid samples under the conditions and purposes described herein.

In Oviedo, on the _____ day of _____________, ________.

Patient's signature:

Investigator's signature:

Place and date:
ID:

Place and date:
ID:

REVOCATION OF INFORMED CONSENT

On ___________________________, I revoke the consent given to participate in the study.

Patient's signature: **Investigator's signature:**

Place and date:

ID:
